# Cis-regulation analysis of RNA m6A methylation and gene expression in colorectal cancer

**DOI:** 10.3389/fgene.2025.1622957

**Published:** 2025-08-14

**Authors:** Zhen-Liang Lin, Pan-Pan Yu, Bei-Lei Ye

**Affiliations:** ^1^ Department of Surgery, The Affiliated Cangnan Hospital of Wenzhou Medical University, Wenzhou, China; ^2^ Department of Gastric Surgery, Zhejiang Cancer Hospital, Hangzhou, China; ^3^ Computational Biology and Bioinformatics Center, Hainan University, Haikou, China

**Keywords:** RNA methylation, m6A, gene expression, colorectal cancer, cis-regulation

## Abstract

RNA N6-methyladenosine (m6A) methylation is a major epigenetic modification that plays a critical role in regulating gene expression in tumors. Although the regulation of individual genes by m6A methylation has been extensively studied, a systematic quantification of transcriptome-wide associations between RNA methylation and gene expression remains limited. In this study, we analyzed publicly available MeRIP-seq and RNA-seq datasets of paired colorectal cancer (CRC) and adjacent normal tissues from four patients, proposing a statistical model to quantify the cis-regulation between m6A methylation and gene expression in CRC. The results indicated that (1) A total of 46,500 and 31,715 unique m6A peaks were identified in CRC and normal control (NC) tissues, respectively. Compared with NC tissues, 538 genes were upregulated and 3,944 were downregulated in CRC tissues (padj <0.05 and |log_2_FC| > 1). (2) Approximately 66.01% of m6A peaks in CRC are located within genes and 28.78% in promoters, compared to 65.00% and 28.38%, respectively, in NC tissues. CRC tissues exhibited higher methylation levels in exons and 3′UTRs, while NC tissues showed increased methylation in introns. (3) 451 genes exhibited significant cis-regulation between RNA methylation and gene expression. Among these, 371 genes were positively correlated, indicating a promotive effect on gene expression, while 80 genes showed negative correlation. Moreover, 34 genes showing strong correlations (*r* ^2^ ≥ 0.9) were identified, including 16 genes previously reported to be associated with CRC. This study provides a transcriptome-wide strategy for quantifying the association between RNA methylation and gene expression in CRC, offering new insights into the potential regulatory roles of RNA methylation in tumor biology.

## 1 Introduction

RNA methylation is one of the key epigenetic modifications involved in biological processes. In recent years, more than 170 types of RNA chemical modifications have been discovered, involving both coding and non-coding RNAs (Wang et al., 2023; Boccaletto et al., 2022). Among these, N6-methyladenosine (m6A) methylation is the most prevalent and biologically significant form of methylation in mammals and has attracted extensive attention (Coker et al., 2019; Xie et al., 2023; Zhang et al., 2020). m6A methylation plays a pivotal role in normal metabolic processes. It has been implicated in a wide array of biological functions including animal development (Frye et al., 2018), regulation of gene expression (Roundtree et al., 2017), emergence of hematopoietic stem/progenitor cells (Zhang et al., 2017), heat shock response (Zhou et al., 2015), neuronal function (Lence et al., 2016), and tumorigenesis (Lan et al., 2021).

Colorectal cancer (CRC), a malignant tumor of the digestive system, is the third most common cancer worldwide and the second leading cause of cancer-related deaths, responsible for over 900,000 deaths annually (https://www.iarc.who.int/cancer-type/colorectal-cancer). Studies predict that by 2030, the number of new cancer cases will exceed 2.2 million, resulting in over 1.1 million deaths (Arnold et al., 2017). By 2040, the global burden of CRC is expected to reach 3.2 million new cases and 1.6 million deaths per year, marking increases of 63% and 73%, respectively (World Health Organization, 2023). Research has shown that the downregulation of some tumor suppressor genes and the overexpression of some oncogenes are closely associated with the onset and progression of colorectal tumors. For instance, *TRAP1* has been found to be highly expressed in CRC tissues, and knocking down *TRAP1* can arrest the cell cycle of CRC cells and promote their apoptosis (Lettini et al., 2016). Ding et al. (2020) reported that *MAD2L1* is significantly overexpressed in CRC tissues than in normal tissues. Knocking down *MAD2L1* can decelerate the cell cycle process, induce cell apoptosis, and directly inhibit tumor growth. Additionally, Watson et al. (2020) investigated age-related differences in gene expression of CRC and found that *PEG10* is more highly expresses in young CRC patients. Furthermore, RNA m6A methylation regulates cellular adaptation to dynamic microenvironments, influencing CRC initiation, progression, and prognosis, while also modulating gene expression to promote proliferation and metastasis (Han, 2024; Jiang et al., 2023).

RNA m6A methylation plays a regulatory role in gene expression, and its effects vary significantly across different species (Luo et al., 2014; Zhang et al., 2021). In former research of CRC, although individual genes affected by methylation have been identified as influencing processes such as tumor occurrence and proliferation, a systematic analysis of the cis-regulation between RNA methylation and gene expression at the gene-level remains to be fully studied. It includes identifying which specific genes are positively regulated by m6A and which genes are negatively affected. To address this gap, we analyzed MeRIP-seq and RNA-seq data from four CRC patients, including central specimens of CRC tissue and paired adjacent normal control (NC) tissues. This study aims to quantify gene-level cis-regulation between RNA m6A methylation and gene expression, provide the dynamic transcriptome-wide view of m6A methylation and gene expression in CRC.

## 2 Materials and methods

### 2.1 RNA methylation and gene expression datasets

All data used in this study were obtained from the Gene Expression Omnibus (GEO) database under accession number GSE190388. This dataset was generated by researchers at Chengdu Medical College and includes paired CRC and adjacent NC tissue samples from four patients. Ethical approval and patient consent were obtained in the original study (Li et al., 2022). The IP libraries using anti-m6A antibody were used to profile RNA m6A methylation, and the input control libraries were used to quantify gene expression. Then datasets of RNA methylation and gene expression were aligned with the latest telomere-to-telomere (T2T) human genome, CHM13v2.0 (https://www.ncbi.nlm.nih.gov/datasets/genome/GCF_009914755.1).

### 2.2 RNA m6A methylations analysis

The quality of raw sequences from Methylated RNA Immunoprecipitation Sequencing (MeRIP-seq) was assessed using FastQC v0.11.9 (https://www.bioinformatics.babraham.ac.uk/projects/fastqc). Adapters and low-quality bases (phred score <20) were trimmed using Trimgalore v0.6.10 with default parameters (https://www.bioinformatics.babraham.ac.uk/projects/trim_galore). HISAT2 v2.1.0 was then used to align reads to the reference genomes of human (Kim et al., 2019). Duplicate reads in the alignment bam files were then marked and removed by MarkDuplicates of Gatk v4.1.3.0 (https://github.com/broadinstitute/gatk), Peaks from each sample was called using MACS2 v2.2.9.1 (https://github.com/macs3-project/MACS) with ‘whole genome’ mode. Transcripts per million (TPM) values, used to measure expression levels for all genes from input samples, were calculated using StringTie v2.2.0 (Pertea et al., 2015), A TPM value of 0.5 was used as the baseline expression threshold for genes of samples, following the European Bioinformatics Institute (EBI)’s guidelines (https://www.ebi.ac.uk/gxa/FAQ.html). Principal component analysis (PCA) of gene expressions across samples was conducted using R package ggplot2 (Wickham, 2016). The distribution of methylation genes was analyzed using R packages ChIPseeker (Yu et al., 2015) and GenomicFeatures (Lawrence et al., 2013).

### 2.3 Differential gene expression analysis

Gene expression count data were extracted using FeatureCounts v2.0 (Liao et al., 2014). Differential expression analysis was conducted to identify differentially expressed genes using R package DESeq2 (Love et al., 2014). Differentially expressed genes were determined based on a threshold of padj <0.05 and |log_2_FC| > 1.

### 2.4 The effect of RNA methylation on gene expression across genes

Considering that gene expression is usually influenced by its own RNA methylation, we focus on the effects of m6A in gene-body on gene expression within the same gene. To explore the specific genes with the effect of m6A on gene expression, the linear regression was used to assess the correlation between gene expression and m6A methylation.

$$Y_{\mathrm{GE}i}=\beta_{i}+B_{i}X_{i}+\varepsilon_{i}$$

Where *Y*_GEi_ represents the Transcripts Per Million (TPM) values of the *i*th (*i* = 1, … , *N*) gene from 8 samples, *Y*_GE*i*_ *=* (*Y*_1*i*_, … , *Y*_8*i*_)^T^; *X*_*i*_ denotes the explanatory variables of methylation level in gene *i*, and *B*_*i*_ represents the corresponding effect coefficients of *X*_*i*_. *β*_*i*_ and ε_*i*_ stand for the intercept term and random error, respectively. Subsequently, we classified the cis-regulation of genes into two groups, positive (+) and negative (−), based on the sign of coefficients. And the value of *r* ^2^ larger than 0.9 was used to obtain the confidence high correlation cis-regulation of the effects of RNA 6 mA methylation on gene expression.

## 3 Results

### 3.1 Profiling of RNA m6A methylation across CRC and NC tissues

**FIGURE 1:**
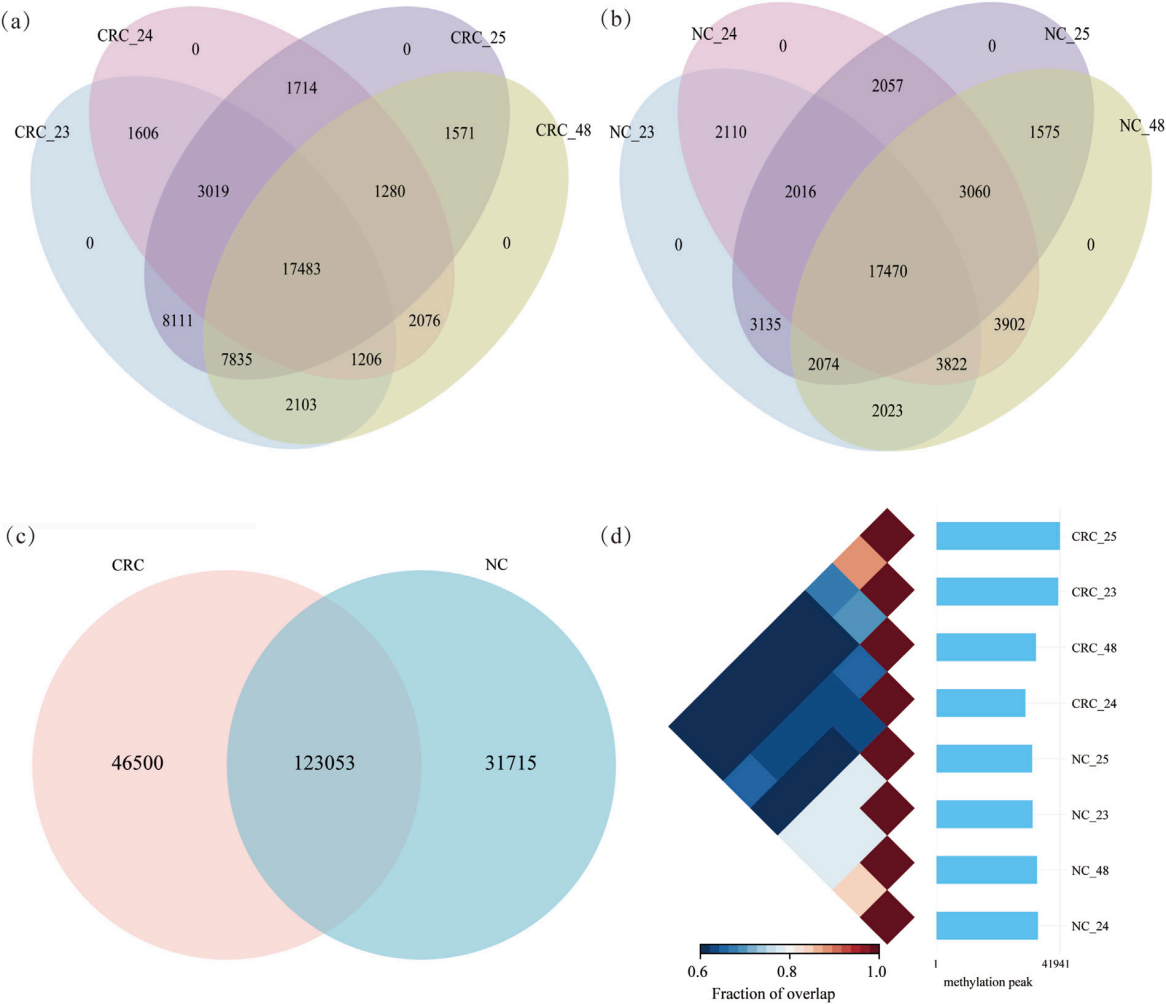
RNA methylation profiling of CRC and NC tissues: **(a)** and **(b)** m6A methylated peaks in CRC and NC tissues, respectively; **(c)** identification of shared and unique m6A peaks between CRC and NC tissues; **(d)** pair relationship between tissues from 8 samples.

The MeRIP-seq datasets from four CRC patients, encompassing two tissues (CRC and NC) were used to profile transcriptome-wide RNA m6A methylation. RNA m6A methylation was captured through immunoprecipitation using m6A-specific antibodies. Each sample comprised paired datasets (IP and input). Clean reads ranged from 5.20G to 7.18G from IP and input samples in CRC patients (Supplementary Table S1). To ensure confidence in m6A peaks for each tissue, peaks identified in at least two replicates were retained (Supplementary Figure S1). In CRC tissues, 41,363, 30,190, 41,941, and 33,791 peaks were identified, respectively, from four replicates. In NC tissues, 32,650, 34,492, 32,507, and 34,141 peaks were identified, respectively (Figures 1a,b; Supplementary Table S2). A total of 169,553 peaks were identified in CRC tissues and 154,768 peaks in NC tissues. Among these, 123,053 peaks were shared between CRC and NC tissues. Unique peaks for CRC and NC tissues were 46,500 and 31,715, respectively (Figure 1c). The confidence of m6A peaks was determined by calculating the proportion of overlapping peaks between any two replicates across eight samples from CRC patients, respectively. Replicates from the same tissues clustered together (Figure 1d), which indicates that the RNA methylation data are robust and suitable for downstream analysis. Additionally, m6A methylated genes identified in at least two replicates were analyzed (Supplementary Figure S2).

### 3.2 Gene expression of CRC and NC tissues

**FIGURE 2:**
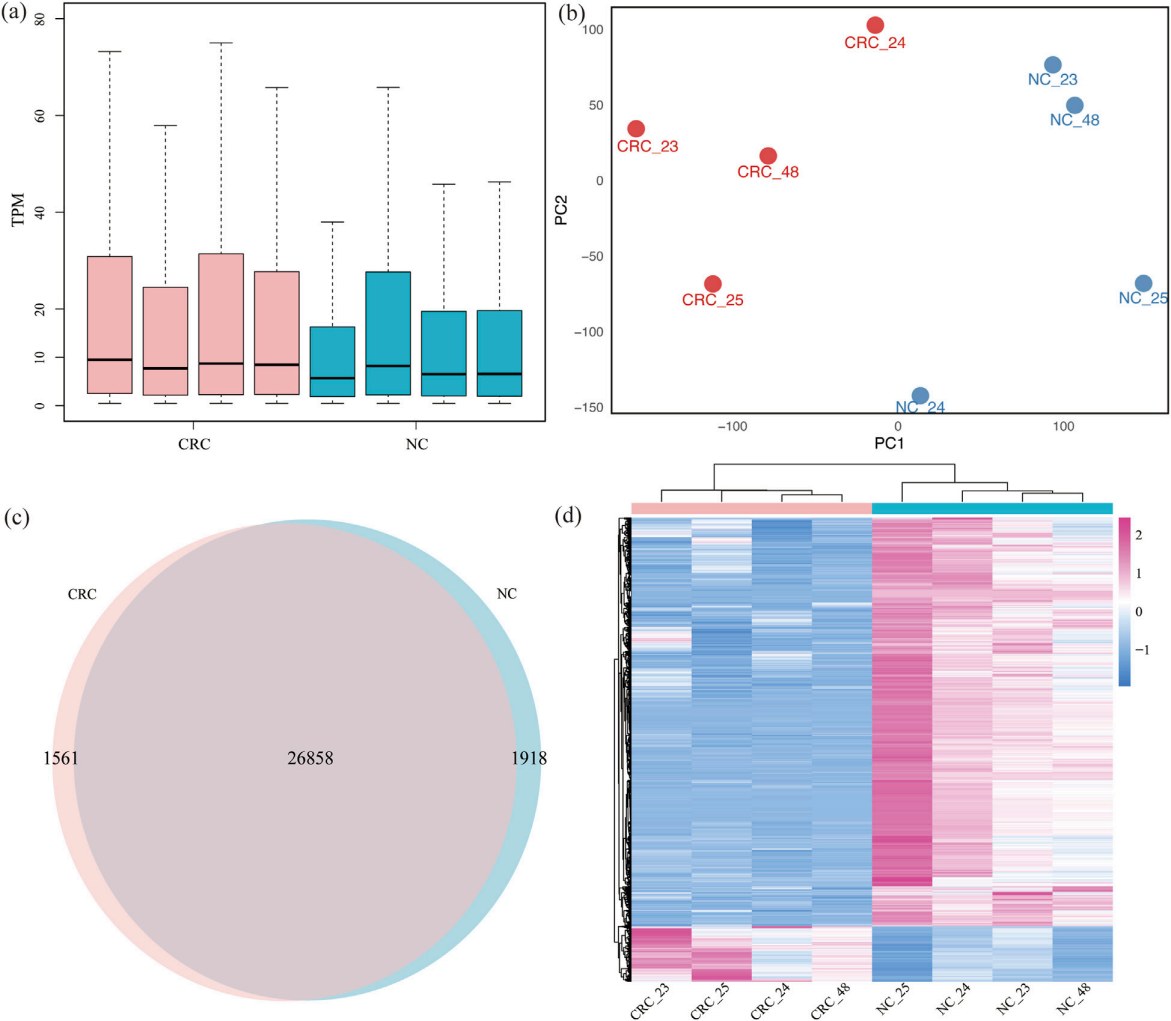
Gene expression profiling of CRC and NC: **(a)** gene RNA expression between CRC and NC across 8 samples; **(b)** the PCA cluster of two kinds of tissues; **(c)** expressed genes in two kinds of tissues; **(d)** heatmap analysis of different genes expression of two tissues.

The RNA sequencing datasets of control samples from MeRIP-seq were utilized to measure the gene expression. The mean of median TPM values for gene expression in CRC and NC tissues were 9.49, 7.72, 8.73, 8.47, 5.70, 8.20, 6.51, and 6.56, respectively. No statistically significant difference was observed between CRC and NC tissues (Figure 2a). Replicates from the same tissues clustered together in principal components analysis (PCA) (Figure 2b), further confirming the reliability of the data used in this study.

A total of 27,070 genes were expressed in both CRC and NC tissues, with 1,561 genes specifically expressed in CRC tissues and 1,918 genes specifically expressed in NC tissues. Among these specifically expressed genes, 25 genes were consistently expressed in all four samples of CRC tissues, while 1,000 genes were consistently expressed in NC tissues (Figure 2c; Supplementary Table S3). This limited number of consistently expressed genes in CRC tissues likely reflects both the intrinsic molecular heterogeneity of CRC and the stringent selection criteria applied in this study. Clustering analysis of differentially expressed genes indicated that CRC and NC samples clustered separately. Using the criteria of padj <0.05 and |log_2_FC| > 1, we identified 4,482 differentially expressed genes, with 538 upregulated and 3,944 downregulated (Figure 2d; Supplementary Table S4).

### 3.3 Genomic features of gene methylation and gene expression

**FIGURE 3:**
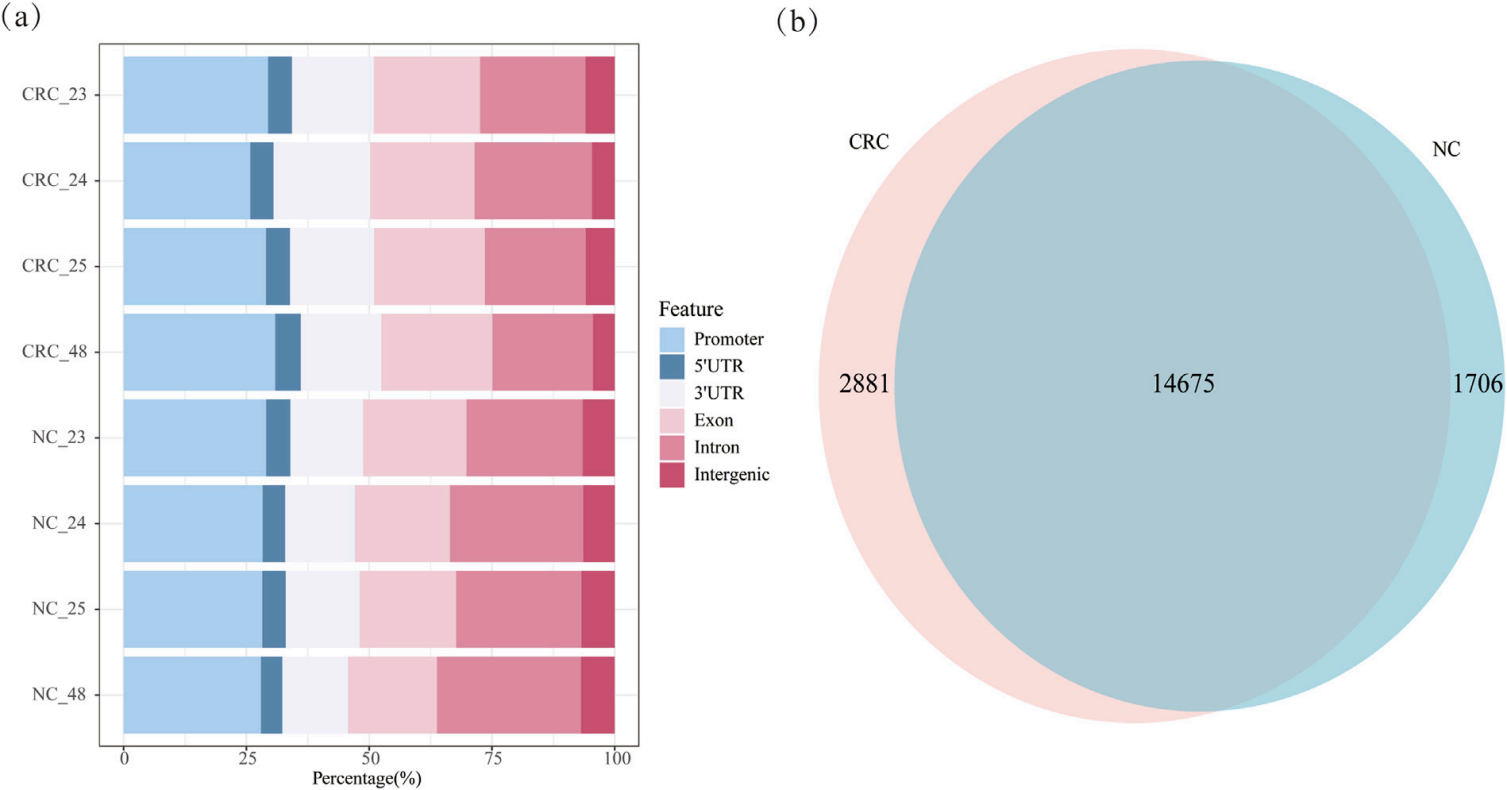
Methylation distribution of CRC and NC tissues: **(a)** m6A methylation distribution of CRC and NC tissues; **(b)** m6A-methylated genes in CRC and NC tissues.

Methylation predominantly localized to different genomic regions. In CRC tissues, methylation was distributed in the promoter (28.78%), exons (22.03%), introns (21.60%), 5′UTR (4.92%) and 3′UTR (17.46%). In NC tissues, 28.39% of methylation was found in the promoter, 26.42% in introns, 19.55% in exons, 4.66% in the 5′UTR and 14.36% in the 3′UTR (Figure 3a; Supplementary Figure S3). The analysis of gene distribution with m6A methylation in both tissue types indicated that over 65% of methylation sites are located within genes, with higher intragenic methylation levels observed in CRC tissues compared to NC tissues. Approximately 66.01% of m6A methylation in CRC occurred within genes and 28.78% in promoters, compared to 65.00% and 28.38%, respectively, in NC tissues. CRC tissues exhibited elevated methylation in exons and 3′UTRs, while NC tissues showed higher methylation in introns. CRC tissues exhibited increased methylation in exons and the 3′UTR, while NC tissues showed higher methylation in the introns. Additionally, 14,675 genes were commonly methylated in both CRC and NC tissues, with 2,881 genes uniquely methylated in CRC tissues and 1,706 genes uniquely methylated in NC tissues, indicating a higher number of methylated genes in CRC tissues (Figure 3b). Further analysis of methylation across the four samples identified 617 genes specifically methylated in all CRC tissues and 342 genes specifically methylated in all NC tissues (Supplementary Table S5). Among the genes methylated in all four CRC samples, several well-characterized genes were observed, including *PDPN*, *MFAP2*, and *RCC1*. *PDPN* encodes a type I transmembrane sialomucin-like glycoprotein that plays a key role in tumor invasion*,* has been reported to be upregulated in cancer-associated fibroblasts (CAFs) in early-stage CRC (Tsukamoto et al., 2024). *MFAP2* was found to be highly expressed and m1A-hypermethylated in CRC, with its overexpression significantly associated with lymph node and distant metastasis, resulting in poor prognosis (Xue et al., 2023). *RCC1* is a guanine nucleotide exchange factor for the Ran GTPase, has been documented that high RCC1 expression in colorectal liver oligometastases is significantly associated with poor recurrence-free and overall survival (Deng et al., 2021).

### 3.4 Cis-regulation between RNA m6A methylation and gene expression

**FIGURE 4:**
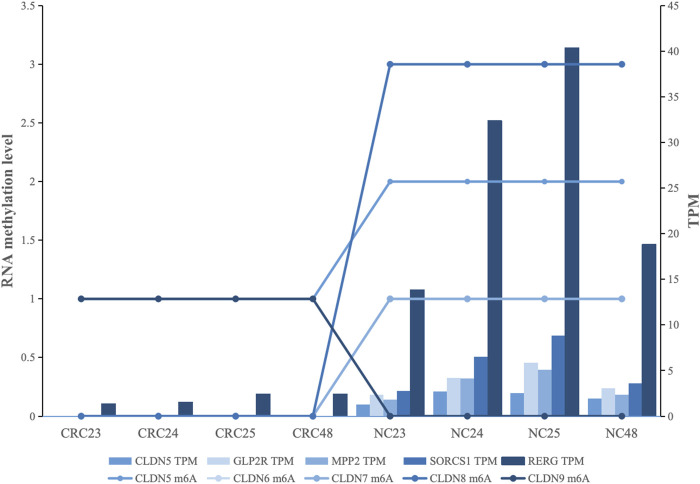
Correlation between RNA methylation and gene expression in CRC-related genes: Each color represents a gene, the bar chart indicates gene expression levels, and the line chart represents the level of methylation sites.

To further study the impact of RNA methylation on gene expression within gene body, we performed linear regression analysis to examine the cis-regulation between RNA m6A methylation within gene body and gene expression for each gene. The cis-regulation of m6A methylation and gene expression within gene were further analyzed by the linear regression. The results indicated that a total of 451 genes were found to have significant associations with RNA methylation. Among these genes, 371 genes showed positive correlation, indicating a promoting effect on gene expression, while 80 genes exhibited a negative effect. This suggests that RNA methylation predominantly exhibited a positive influence on gene expression, accounting for 82.26% of total RNA methylation (Supplementary Table S6). In addition, we obtained the high correlation cis-regulation for the effect of m6A methylation on gene expression with *r* ^2^ ≥ 0.90. A total of 34 genes were identified, among which 85.29% displayed a positive correlation, indicating a predominant enhancing effect of m6A methylation on transcription (Supplementary Table S7). Notably, 16 genes (*CLDN5*, *GLP2R*, *TMPRSS6*, *MPP2*, *SorCS1*, *CNGA3*, *MYO3A*, *SEMA3E*, *ABCB5*, *ASPA*, *NRG2*, *SEMA3D*, *SLC11A1*, *RERG*, *VPS45*, *CBX6*) have been reported to be associated with CRC development (Hua et al., 2017; Guo et al., 2018; Sun et al., 2020; Alghamdi and Al-Zahrani, 2023). From these, we further selected the top five genes with the highest r^2^ values (*GLP2R*, *MPP2*, *SorCS1*, *RERG*, and *CLDN5*) for detailed visualization (Figure 4). In *GLP2R*, *MPP2*, and *SorCS1*, there was a positive correlation between RNA methylation and gene expression, whereas in *RERG*, a negative correlation was observed between RNA methylation and gene expression. *CLDN5* is an essential component of the tight junction proteins, and its expression is associated with the clinical features and prognosis of CRC patients. In our research, *CLDN5* expression is positively correlated with RNA methylation (Figure 4).

## 4 Discussion

RNA m6A methylation is the most prevalent and extensively studied mRNA modification. Recent studies have not only unveiled the molecular mechanisms involved in m6A methylation but also highlighted its critical role in regulating gene expression and various biological processes (He and He, 2021). m6A methylation has been identified as a critical regulator of oncogenes and tumor suppressor genes in cancer, impacting various aspects of cancer progression including tumor initiation, growth, and metastasis. This study established a statistical model to systematically analyze the correlation between m6A methylation and gene expression in CRC at a genome-wide level.

Elevated levels of m6A methylation can enhance the stability and translational efficiency of oncogenes, thus promoting the proliferation and survival of cancer cell (Lan et al., 2019). Previous studies have revealed that gene expression and RNA methylation levels in CRC tissues differ from those in NC tissues (Liang et al., 2022). In this study, we observed a significant increase in RNA m6A methylation modification in CRC tissues compared to NC tissues, along with higher intragenic methylation levels. Through linear regression analysis, we identified 34 genes with high-correlation interactions between RNA methylation and gene expression, among which 16 genes (*CLDN5*, *GLP2R*, *TMPRSS6*, *MPP2*, *SorCS1*, *CNGA3*, *MYO3A*, *SEMA3E*, *ABCB5*, *ASPA*, *NRG2*, *SEMA3D*, *SLC11A1*, *RERG*, *VPS45*, *CBX6*) have been reported to be associated with CRC. This underscores the significant role of specific gene RNA methylation in the regulation of gene expression within CRC. Yang Hua et al. noted decreased *SorCS1* expression in colorectal cancer cells. *ABCB5* plays critical role in CRC development, potentially regulating CRC aggressiveness by enhancing the AXL signaling pathway (Guo et al., 2018). Moreover, SEMA3E signaling pathway has been shown to impact the invasion and metastasis of CRC (Hagihara et al., 2022). These findings suggest that our statistical model accurately reflects the correlation between RNA methylation and gene expression in CRC. In this study, *SorCS1*, *ABCB5*, *NRG2*, *GLP2R*, *MYO3A*, *ASPA*, and *SEMA3E* were not expressed in CRC tissues, with a positive correlation between RNA methylation and gene expression, indicating that low methylation levels of these genes might inhibit CRC progression. Abnormal expression of RERG is associated with tumorigenesis, progression, and metastasis, with downregulation observed in several cancer types, including colorectal cancer (Yang et al., 2014; Xiong et al., 2019). Our results show that the RNA methylation of *RERG* is negatively correlated with its gene expression, implying that high m6A methylation of *RERG* may promote CRC.

In addition to their correlation with CRC gene expression and m6A methylation patterns, several candidate genes identified in our integrative analysis have been previously reported to be associated with the diagnosis and prognosis of CRC. Notably, *SEMA3E* was identified as a key prognostic gene using machine learning approaches, with its high expression levels linked to distinct immune subtypes and poor clinical outcomes in CRC, suggesting its potential as a diagnostic and prognostic biomarker (Zhu et al., 2025). *GLP2R* has been identified as a significantly downregulated gene in colorectal cancer and is strongly associated with immune cell infiltration. Importantly, low *GLP2R* expression correlates with poor overall survival, highlighting its potential as a prognostic biomarker (Maurya et al., 2023). High mRNA expression of *CBX6* was associated with short overall survival in rectal cancer patients. It could be potential prognostic biomarkers for the survival of CRC patients (Li et al., 2020). SLC11A1 has been identified as a predictive biomarker for overall survival in CRC, with high expression consistently associated with poorer prognosis across multiple independent cohorts. Its tumor-specific upregulation and involvement in immune microenvironment modulation further support its potential clinical utility (Hsu, 2024). MPP2, a membrane-associated guanylate kinase, has recently been identified as a potential prognostic biomarker in CRC, with its expression negatively regulated by DNA methylation. Although not directly linked to m6A modification yet, its association with immune infiltration and cell proliferation suggests that epigenetic mechanisms, including m6A, may contribute to its regulatory network and functional relevance in CRC (Yang et al., 2024). Other genes such as *ABCB5*, *CLDN5*, *RCC1* have also been implicated in CRC-related processes, with several studies suggesting their potential value in tumor diagnosis, progression monitoring, or prognostic evaluation (Alghamdi and Al-Zahrani, 2023; Norhisan et al., 2024; Deng et al., 2021). These findings are concordant with our computational predictions, providing indirect yet substantial evidence for the biological and clinical relevance of the m6A-associated genes identified in this study. Future experimental studies are warranted to elucidate the underlying regulatory mechanisms and to validate their clinical utility in CRC diagnosis, prognosis, and therapeutic targeting.

Among the 34 genes, several have not been previously reported in association with CRC, such as *GPRASP2*, *TSBP1*-*AS1*, *ASXL3*, and *TDRD10*. These genes may represent novel CRC-associated candidates and contribute to the m6A-mediated transcriptomic regulation. *GPRASP2* encodes a G protein-coupled receptor-associated sorting protein and has been implicated in vesicle trafficking and GPCR recycling pathways in neurological tissues (Simonin et al., 2004). Although its role in cancer is unclear, GPCR signaling has been widely recognized for its involvement in tumor proliferation and metastasis (Lappano and Maggiolini, 2012), suggesting *GPRASP2* may participate in oncogenic pathways via m6A-related post-transcriptional regulation. *ASXL3* is an epigenetic regulator that participates in chromatin remodeling and transcriptional control. Recent studies have highlighted its oncogenic roles in small cell lung cancer, where it promotes tumor cell proliferation and survival. Although its role in CRC has not been reported, its strong correlation with m6A methylation suggests it may contribute to CRC progression through epigenetic and post-transcriptional regulatory mechanisms. *TDRD*, a conserved protein family associated with RNA metabolism and germ cell development due to its binding to methylarginine, have been detected in various cancers. Previous study has identified differential expression of *TDRD5*, *TDRD6*, and *TDRD7* in CRC (Fan et al., 2020). In this study, the RNA methylation of *TDRD10* were positively correlated with gene expression. Although no direct link between *TDRD10* and CRC has been reported, *TDRD10* has been identified as a promising diagnostic and prognostic marker for breast cancer and other cancers (De Almeida et al., 2019), meriting further investigation in CRC. *TSBP1-AS1* is a lncRNA with no prior reports in CRC. However, several antisense lncRNAs have been shown to modulate m6A methylation and act as oncogenic drivers or suppressors. These findings suggest that unreported but m6A-correlated genes may play functionally relevant roles in CRC and deserve further functional investigation. Our data provide a resource for exploring novel m6A-related regulatory mechanisms in tumorigenesis.

Recent studies have demonstrated that m6A methylation modulates gene expression by influencing mRNA stability, splicing, and translation through specific reader proteins such as YTHDF1/2/3 (Wang et al., 2014; 2015) and IGF2BP1/2/3 (Huang et al., 2018). Although our study did not directly explore these molecular mechanisms, the observed strong correlations between m6A methylation and gene expression suggest that these regulatory pathways may also be involved in CRC progression. Further functional validation of the involvement of these m6A reader-mediated mechanisms in CRC would be a valuable direction for future research.

This study employs a linear model to examine the cis-regulation between RNA methylation and gene expression in CRC, unveiling dynamic genome-wide interactions between RNA methylation and gene expression. Several novel CRC-related candidates were identified, suggesting new avenues for understanding epitranscriptomic regulation in CRC. The quantitative method established for the relationship between RNA methylation and gene expression can be applied to other studies of tumor datasets and can also be extended to study on the correlation between RNA methylation and gene expression in plants and animals. The findings of this study serve as a reference for analogous studies involving tumor-related datasets, furnishing crucial backing for the advancement of precision medicine treatments grounded on RNA methylation.

## Data Availability

The original contributions presented in the study are included in the article/Supplementary Material, further inquiries can be directed to the corresponding authors.
