# Supplementary material for: Cis-regulation analysis of RNA m6A methylation and gene expression in colorectal cancer
Source: Front Genet. 2025 Aug 14;16:1622957. doi: 10.3389/fgene.2025.1622957 (PMC12392630; doi:10.3389/fgene.2025.1622957)
Supplement: Supplementary file 2 [file DataSheet1.docx]

Supplementary Figures

##
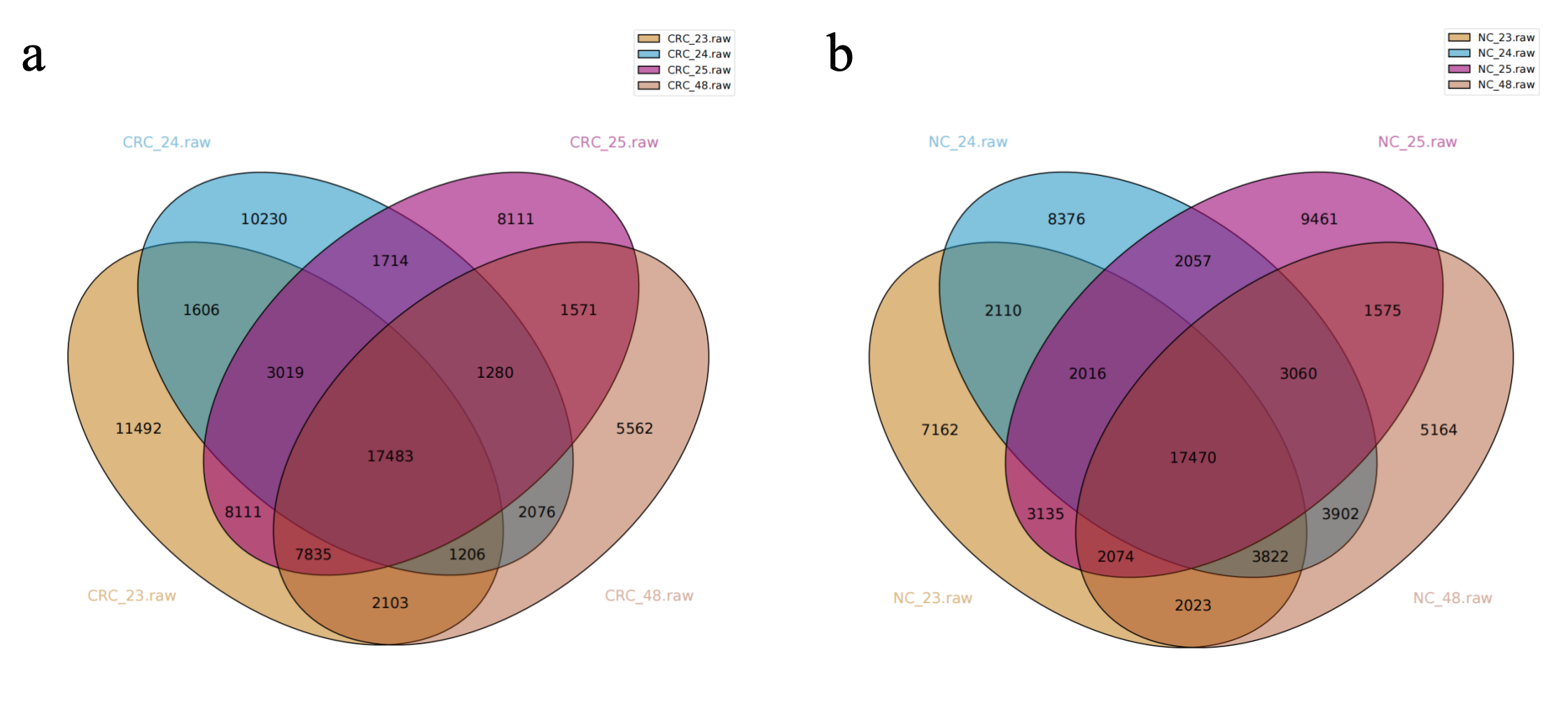


**Supplementary Figure 1.** **a and b** m6A peaks identified in each sample of CRC and NC tissues


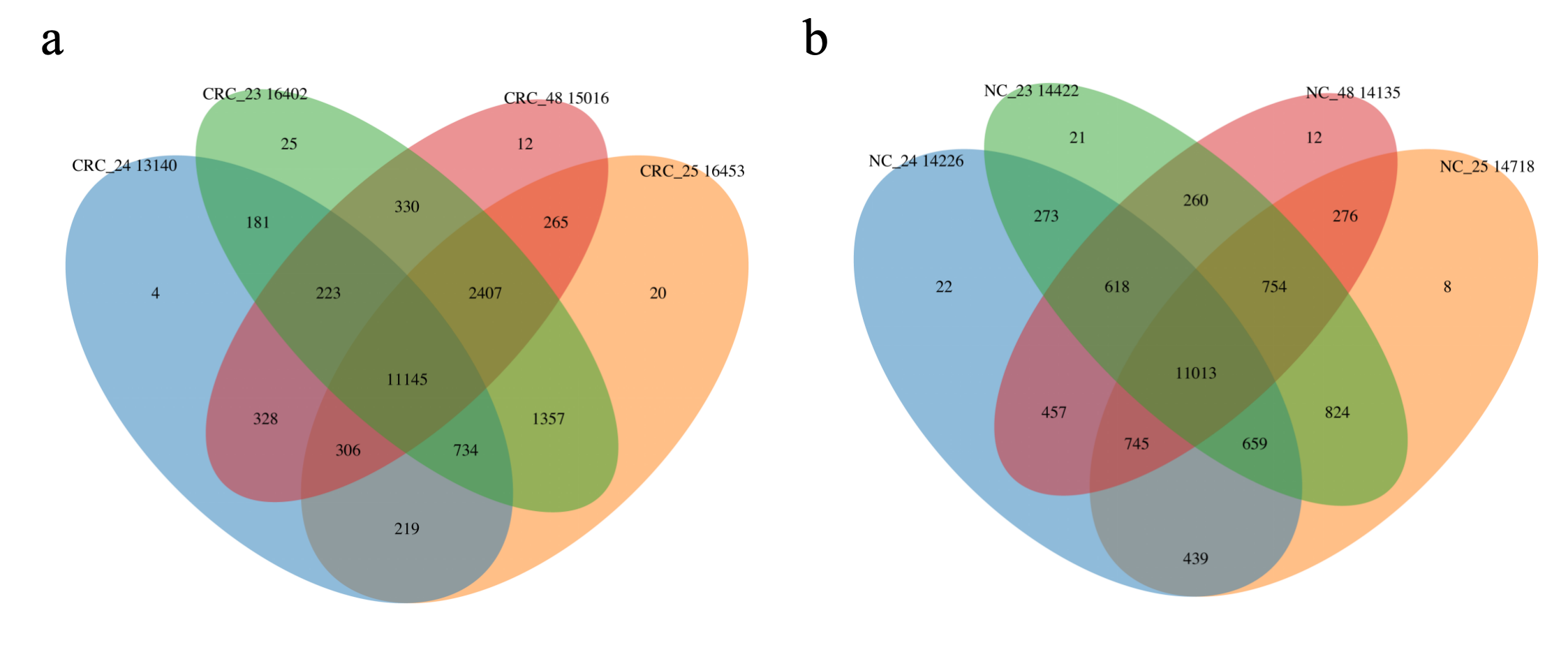


**Supplementary Figure 2. a and b** m6A methylated genes identified in at least two replicates of CRC and NC tissues


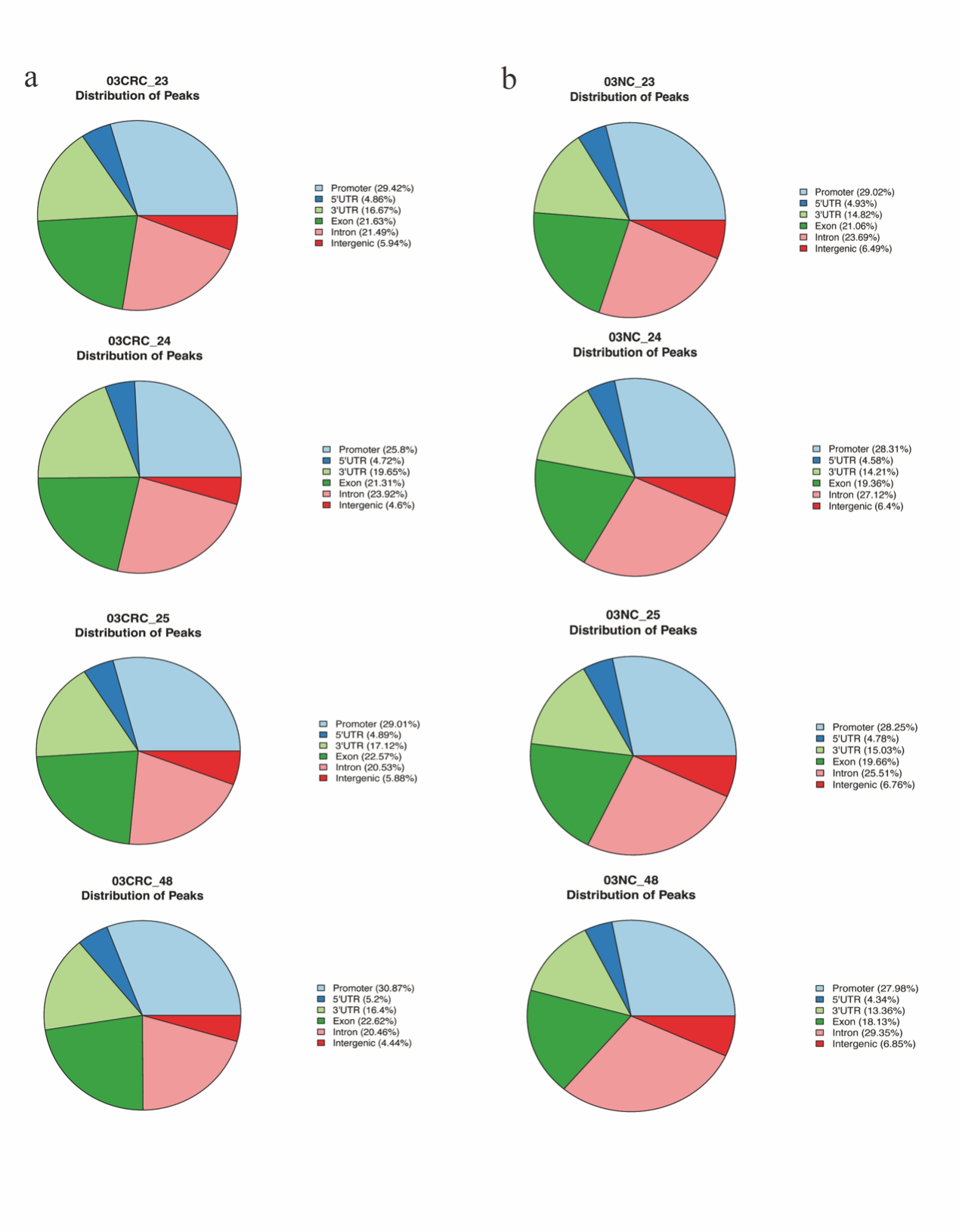


**Supplementary Figure 3. a and b** Genomic distribution of m6A peaks in CRC and NC tissues
